# Associations between preference and participation in team sports: Physical activity promotion among adolescents

**DOI:** 10.3389/fpubh.2022.1024932

**Published:** 2022-11-29

**Authors:** Karel Frömel, Josef Mitáš, Dorota Groffik, Michal Kudláček, Pavel Háp

**Affiliations:** ^1^Faculty of Physical Culture, Palacký University Olomouc, Olomouc, Czechia; ^2^Institute of Sport Science, The Jerzy Kukuczka Academy of Physical Education, Katowice, Poland

**Keywords:** physical activity recommendations, sports preferences, IPAQ, soccer, volleyball

## Abstract

**Background:**

The level of physical activity (PA) in adolescents is highly dependent on their PA preferences. PA preferences among adolescents are dominated by team PA, mostly team sports (TS). The aim of this study is to identify (a) the status and trends in the preferences of TS among Czech and Polish boys and girls in different educational and sports environments, and (b) the impact of the agreement between the preferred and simultaneously pursued TS on the structure of weekly PA and on the meeting of PA recommendations.

**Methods:**

The research was carried out between 2009 and 2022 in the Czech Republic and Poland and included 2,939 boys and 4,427 girls aged 15–19 years. Preferences and participation in TS were identified using a PA preference questionnaire and weekly PA using the International Physical Activity Questionnaire-Long Form. Trends in TS preferences were analyzed over 27-year periods. The participants were divided into groups by agreement and disagreement between the preferred and pursued TS, and by agreement and disagreement between the preferences of TS and participation in organized TS.

**Results:**

Throughout the 14-year study period, boys in both countries preferred soccer, whereas girls favored volleyball. Agreement between preferences and participation in TS increased vigorous PA in Czech and Polish boys and girls. The agreement between the preferences for TS and participation in organized TS had the most significant effect on increasing vigorous PA in Czech and Polish boys and girls and on total PA in boys in both countries. Those who preferred and participated in TS were more likely to meet PA recommendations.

**Conclusion:**

Respecting the status and trends of TS preferences in supporting participation in TS increases adolescents' PA and their achievement of PA recommendations. Increasing active participation in organized TS among boys and girls may support regular PA and help eliminate the negative effects of the pandemic on adolescents' PA.

## Introduction

Team physical activity (PA) is based on team cooperation and is mostly presented in studies as team sports (TS), group sports (1) or collective sports (2). In this study, we lean toward the term team sports, which we understand as group PA of competitive and non-competitive nature, performance and recreational, following and not following rules, with and without a group leader. TS understood in this way is one type of PA.

TS has numerous benefits for adolescent health (3) and has the potential to increase adolescent PA levels (4). According to Oliveira et al. (1), TS may improve body composition, cardiorespiratory endurance, and handgrip strength in overweight/obese youth. The psychological and social benefits of participating in TS are convincing. Participation in TS supports group cohesion and awareness of the individual roles in the group, which is an important aspect of socialization (5). Unlike individual sports, participation in TS may provide greater protection for adolescents from depressive symptoms (6) and may also lead to better mental health in adulthood (7). According to a large study conducted in the USA, TS is strongly associated with improvements in mental health (8). Participation in TS may strongly decrease loneliness (9).

In addition to the positive effects of participation in TS, one must be aware of the negative side effects. These include a higher risk of injury compared to individual sports (10–12), antisocial behavior toward out-groups (13), unhealthy behavior such as smoking or harmful substance use (6, 14, 15), and other manifestations of immoral behavior. Physical education and the adoption of health and physical literacy play an irreplaceable role in eliminating the possible occurrence of these negatives associated with participation in TS (16). However, concerning aspects of family life, simplified forms of TS may have significant intergenerational effects on sports education, as well as on a physically active lifestyle.

Monitoring and analyzing the changes in preferences and participation in TS are extremely important during the pandemic. This is especially important because the pandemic has been associated with a significant decrease in PA (17–20), negative impacts on mental health (21, 22), and a decrease in adolescents' fitness (23). The restrictions on organized PA (OPA) had a significant negative effect on the habit of regular PA (24, 25).

However, the solution to these serious issues is also dependent on the theoretical background of preference diagnosis. Unfortunately, the theory of PA preferences has not been sufficiently elaborated despite being the basis for choosing the strategy for optimal participation in PA and promotion of adolescents' healthy lifestyles. This cannot be justified by the fact that existing preference theories have been criticized in other fields (26, 27). There is an absence of a theoretical background for the assessment of differently conceived preferences, inclinations, favoring motivations, or interests. In this study, we understand PA preferences as a set of offers of PA types that will allow continuous evaluation of their exogenous and endogenous current and future personal benefits. In the concept of PA preferences, we especially respect the findings from the global record of economic preferences (28). Similarly, the reliability of preference research methods is insufficient (29, 30). Therefore, we aimed to improve the theoretical background of research on adolescents' PA through continuous and parallel research on their preferences and participation in TS. The analysis of associations between PA preference and performance can bring new insights into research on the stability of PA preferences and changes in adolescent physical behavior. Our focus was on whether the consensus between preferred and pursued TS supports PA and the achievement of PA recommendations among adolescent boys and girls. In doing so, we took into account the important role of participation in organized TS. We consider structured PA with a clear focus and under the guidance of a qualified “instructor” to be organized TS.

This study aimed to ascertain (a) the status and trends in TS preferences among boys and girls in different educational and sports environments in the Czech Republic and Poland, and (b) the impact of the agreement between the preferred and simultaneously pursued TS on weekly PA and PA recommendations.

## Materials and methods

### Participants and settings

This research was conducted between 2009 and 2022 in 90 Czech and 57 Polish secondary schools. There are similar types of 4-year secondary schools in both countries, and they do not differ significantly in school curricula. In Poland, however, schools have four classes of physical education per week (in the Czech Republic, usually only two) and more emphasis is placed on school sports. The research in the Czech Republic was coordinated throughout by the Palacký University in Olomouc and in Poland by the Academy of Physical Education in Katowice. Every year, we approached 5–10 schools in both countries cooperating with these universities. On average, 85–95% of schools agreed to participate on the research. Only in the years 2021–2022, 30% of the schools could not participate in the research in both countries. During the selection, we considered the type of region, the type of school and we also requested that schools had not previously participated in similar research. Each year, 400–650 participants took part in the research. On average, more than 90% of the approached students participated in the research. All schools, teachers, parents of students, and individual students were involved in accordance with their consent to participate. Other inclusion criteria were type of region, type of school, and previous non-participation in the research. Each year, the study involved 400–650 participants. In total, the research in the Czech Republic involved 1,684 boys (age = 16.8 ± 1.2 years, weight = 70.4 ± 11.7 kg, height = 178.8 ± 8.0 cm, BMI = 22.0 ± 3.1 kg·m^−2^) and 2810 girls (age = 16.9 ± 1.2 years, weight = 59.1 ± 9.0 kg, height = 167.2 ± 6.6 cm, BMI = 21.1 ± 2.8 kg·m^−2^). In Poland, the research involved 1,255 boys (age = 16.4 ± 0.9 years, weight = 68.1 ± 12.3 kg, height = 177.2 ± 7.5 cm, BMI = 21.6 ± 3.3 kg·m^−2^) and 1,617 girls (age = 16.3 ± 0.8 years, weight = 57.2 ± 9.0 kg, height = 165.85 ± 6.1 cm, BMI = 20.8 ± 2.9 kg·m^−2^). Only 14.3% of the boys and 8.6% of the girls in the entire sample reported being overweight (≥25 kg·m^−2^). The participants were divided into groups by agreement and disagreement between the preferences of TS and participation in TS and by agreement and disagreement between the preferences of TS and participation in organized TS. The introductory session concerning the completion of the questionnaires was held in a school computer lab during classes, under supervision of the same research teams and the responsible representative of the school. All participants were registered filled in first the International Physical Activity Questionnaire and the second was the Questionnaire on Preference of Physical Activity in the web application “International Database for Research and Educational Support” (Indares) (www.indares.com).

### Measurements

#### Preferences of team sports

The preferences for TS were identified by the Questionnaire on Preference of Physical Activity, which is regularly used in the Central European region as part of the web-based application Indares. The questionnaire has been standardized for the Czech Republic and Poland (31, 32), and includes the following types of PA: individual, team, fitness, water, outdoors, martial, and rhythmic dance. Team PA includes the following: football, baseball (softball), basketball, curling, floorball (field hockey, unihockey, etc.), soccer (futsal), frisbee, handball (dodgeball), lacrosse, ice hockey (in-line hockey), football tennis, rugby, water polo, and volleyball (beach, catch). Any PA types not specified in the list or new PA types were classified by respondents under the closest PA type or under the “others” item.

The respondents chose the first five preferred PA types that received points according to their ranking. The unselected PA types received the average score of the remaining rankings. The order of preference was determined by the sum of the points scored. The questionnaire also asked about the most frequent leisure PA during the past 12 months and active participation in regular PA (under the supervision of a coach, teacher, trainer, or another leader) during the past 12 months. In this study, only those PA type preferences ranked first were considered.

#### Subjective assessment of weekly physical activity

The Czech and Polish version of the “International Physical Activity Questionnaire-Long Form” was used in the Indares web-based application (33, 34). Both versions were translated according to the EORTC Quality of Life Group (35). The Pearson's correlation coefficient of concurrent validity between overall weekly PA (METs-min) and weekly step counts ranged from *r* = 0.231 to 0.283. Cronbach's alpha, an indicator of internal consistency reliability, was 0.848 for the Polish version and 0.845 for the Czech version. The questionnaire structures PA based on type (job/school-related PA; transportation PA; housework, house maintenance, and caring for family; recreation, sport, and leisure-time PA), intensity (vigorous, moderate, and walking), and time spent sitting. Higher estimates of PA time (36) and lower estimates of time spent sitting were partly eliminated by questionnaire modifications, which allowed us to maintain the highest possible degree of objectiveness of the PA structure. Contrary to the questionnaire manual, the MET-min of vigorous PA was evaluated using a multiple of six (instead of eight). The permissible maximum average daily sum of PA and transport minutes was set at 960 min/day, and the maximum number of MET-min/week was set at 16,000 MET-min/week. A total of 234 respondents were excluded because they did not comply with above mentioned predetermined criteria.

Weekly PA recommendations were determined according to the Healthy People 2030 guidelines (37). The questionnaire allows recommendations to be determined according to a single type of moderate-to-vigorous PA (MVPA) or a single type of vigorous PA (VPA). Therefore, the recommendation of at least 60 min of MVPA was decreased for 5 or more days a week, and concurrently, at least 20 min of VPA 3 or more times per week (38).

We recorded and analyzed the data in the Indares web application.

### Statistical analysis

Data were analyzed using Statistica version 14 (StatSoft, Prague, Czech Republic) and SPSS version 25 (IBM, Armonk, NY, IBM Corp.). Descriptive characteristics were used to check eligibility for statistical analyses and to determine the ranking of preferences and participation in TS. Group differences were determined using crossing tables and the Kruskal–Wallis ANOVA test. Unfortunately, the requirements for the use of multilevel regression analysis (39) were not met, particularly because of the non-normal distribution of the dependent variables and the multicollinearity of the independent variables. In the analysis of the likelihood of meeting the VPA recommendations, the criteria for using a bilogistic regression analysis were met. Due to the higher number of categorical variables, we used forward stepwise methods in the binary logistic regression analysis. The following were included as control variables: age (<17 or ≥ 17 years), cottage use (no–yes), car use (no–yes), home (house or flat), dog ownership (no–yes), and bicycle ownership (no–yes). We selected these control variables from 17 possible ones based on their importance in previously conducted studies (32, 38), on the possibility of coding categorical variables, and on meeting other criteria required for the use of binary logistic regression (40, 41). To determine practical significance, we used the “effect size” coefficients η^2^ (0.01 ≤ η^2^ < 0.06 small; 0.06 ≤ η^2^ < 0.14 medium; and η^2^ ≥ 0.14 large effect size) and *r* (0.1 ≤ *r* < 0.2 small; 0.2 ≤ *r* < 0.6 medium; and *r* ≥ 0.6 large effect size). The level of significance was set at *p* ≤ 0.05 (39, 42).

## Results

### Status and trends in TS preferences

Team sports were the most preferred type of sports among Czech and Polish boys throughout the 14-year monitoring period. In total, TS was preferred by 43.5% of the Czech boys and 39.9% of the Polish boys. The most preferred TS among Czech (29.2%) and Polish (41.4%) boys was soccer within all the individual time periods (Table 1). Czech boys also preferred floorball (17.3%), basketball (9.6%), volleyball (8.5%), and ice hockey (7.6%). Polish boys preferred volleyball (19.1%), basketball (14.6%), or handball (6.1%). The main difference between Czech (17.3%) and Polish (1.6%) boys was in the preference for the floorball (χ^2^ = 187.01, *p* < 0.001, *r* = 0.252).

**Table 1:** Trends in the preferences of team sports among Czech (CZ) and Polish (PL) boys and girls from 2009 to 2022 (first place preferences).

**Time period**	**2009–2010**	**2011–2012**	**2013–2014**	**2015–2016**	**2017–2018**	**2019–2020**	**2021–2022**
Boys CZ %	Soccer 39.1	Soccer 27.1	Soccer 27.4	Soccer 31.5	Soccer 22.5	Soccer 24.9	Soccer 28.7
Boys PL %	Soccer 44.5	Soccer 46.9	Soccer 45.2	Soccer 42.7	Soccer 40.9	Soccer 37	Soccer 32.1
Girls CZ %	Volleyball 41.8	Volleyball 35.9	Volleyball 32	Volleyball 35.6	Volleyball 40.8	Volleyball 31.7	Volleyball 30.9
Girls PL %	Volleyball 62.2	Volleyball 49.7	Volleyball 43.4	Volleyball 46.2	Volleyball 52.3	Volleyball 35.3	Volleyball 37.6

In total, 24.1% of Czech girls and 25.7% of Polish girls preferred TS. Czech (35.9%) and Polish girls (45.6%) clearly preferred volleyball within the monitored 2-year time periods (Table 1). The Czech girls also preferred basketball (12.2%), handball (10.8%), floorball (9.5%), and soccer (7.6%). Polish girls preferred basketball (18.7%), handball (9.3%), and soccer (7.1%). Significant differences between Czech and Polish girls were found in the preference for basketball (χ^2^ = 34.71, *p* < 0.001, *r* = 0.088) and floorball (χ^2^ = 83.51, *p* < 0.001, *r* = 0.137).

Increased attention must be paid to the fact that, compared with the first stage of the research (2009–2010), in the last research stage (2021–2022) during the pandemic, an increase was observed in the preferences of individual PA (χ^2^ = 43.67, *p* < 0.001, *r* = 0.149); in Czech boys (21.9–32.6%, *p* = 0.020), Polish boys (11.8–32.2%, *p* < 0.001), Czech girls (19.2–23.1%, *p* = 0.037), and Polish girls (22.2–32.6%, *p* = 0.038).

### Agreement between preferences and participation in TS in vigorous and total weekly PA

**Figure 1:**
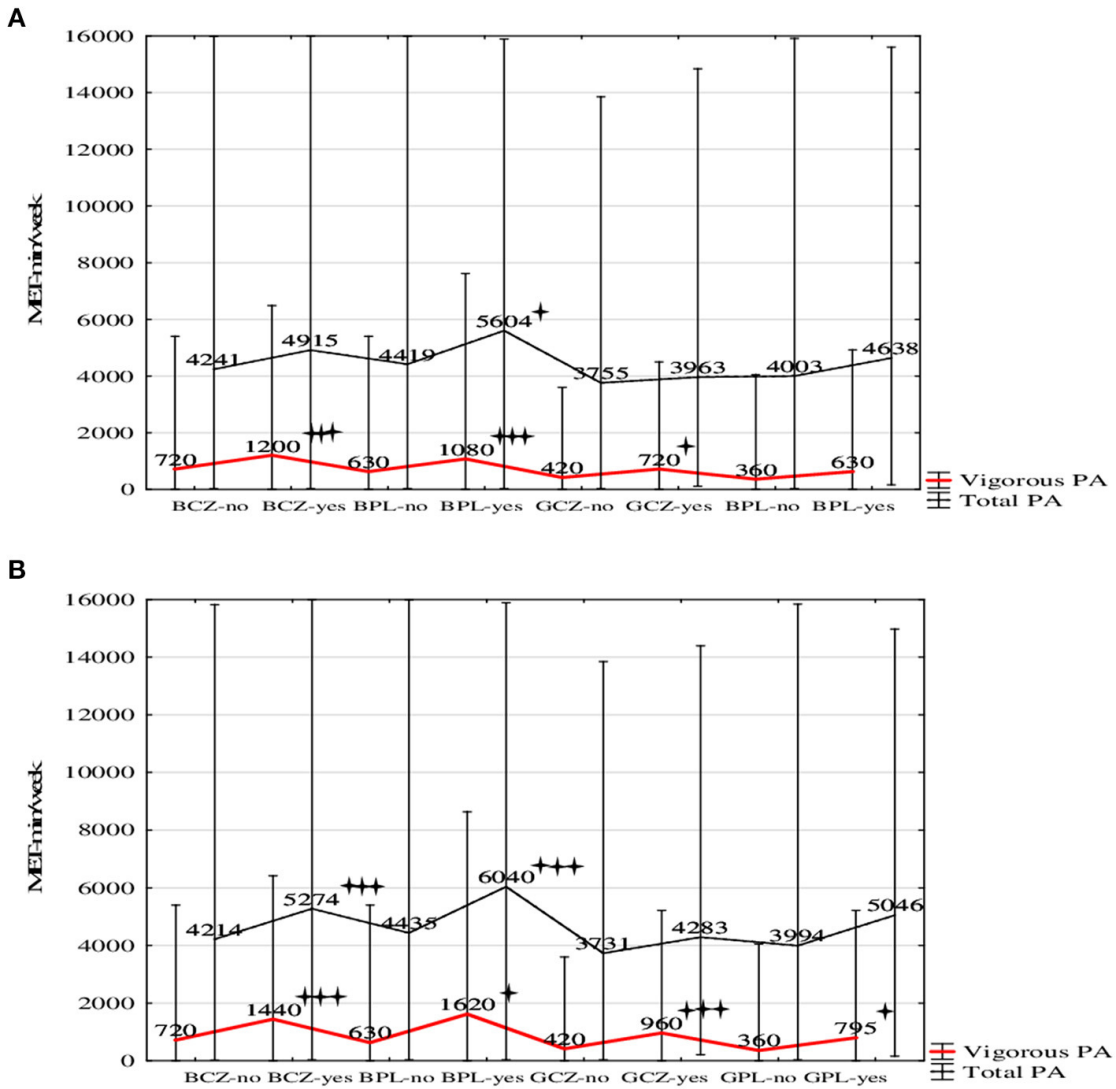
Weekly PA (Mdn) in Czech boys (BCZ) Polish boys (BPL), Czech girls (GCZ) and Polish girls (GPL) by the agreement between the preferences and participation in team sports (yes–no) **(A)** and preferences and participation in organized team sports (yes–no) **(B)**.

The agreement between the preferences and participation in TS was associated with higher vigorous PA (*H* = 178.18, *p* < 0.001, η^2^ = 0.023) in Czech boys (*p* < 0.001), Polish boys (*p* < 0.001), and Czech girls (*p* = 0.018) (Figure 1A) and with total weekly PA (*H* = 61.69, *p* < 0.001, η^2^ = 0.007) only in Polish boys (*p* = 0.030).

The agreement between the preferences for TS and participation in organized TS was significantly associated with higher weekly VPA (*H* = 244.52, *p* < 0.001, η^2^ = 0.032) in Czech boys (*p* < 0.001), Polish boys (*p* = 0.018), Czech girls (*p* < 0.001), and Polish girls (*p* = 0.030; Figure 1B); in total weekly MVPA (*H* = 94.95, *p* < 0.001, η^2^ = 0.012) in Czech (*p* < 0.001) and Polish (*p* < 0.001) boys only.

The agreement between the preferences for TS and participation in organized TS was reflected in school PA (*H* = 111.41, *p* < 0.001, η^2^ = 0.014) in Polish boys (*p* = 0.004) and Polish girls (*p* < 0.001), and in recreational PA (*H* = 161.26, *p* < 0.001, η^2^ = 0.021) in Czech boys (*p* < 0.001) and Czech girls (*p* = 0.003). A remarkable finding was that the agreement between the preferences and participation in TS was significantly associated (*H* = 25.57, *p* < 0.001, η^2^ = 0.003) with a decrease in the average daily sitting time in Czech (*p* < 0.001) and Polish (*p* = 0.041) boys, but not in girls. Similarly, the agreement between the preferences and participation in organized TS (*H* = 31.12, *p* < 0.001, η^2^ = 0.004) was reflected in Czech (*p* < 0.001) and Polish (*p* = 0.005) boys, but not girls.

### Meeting PA recommendations by agreement between the preferences of TS and participation in TS and by agreement between the preferences of TS and participation in organized TS

**Figure 2:**
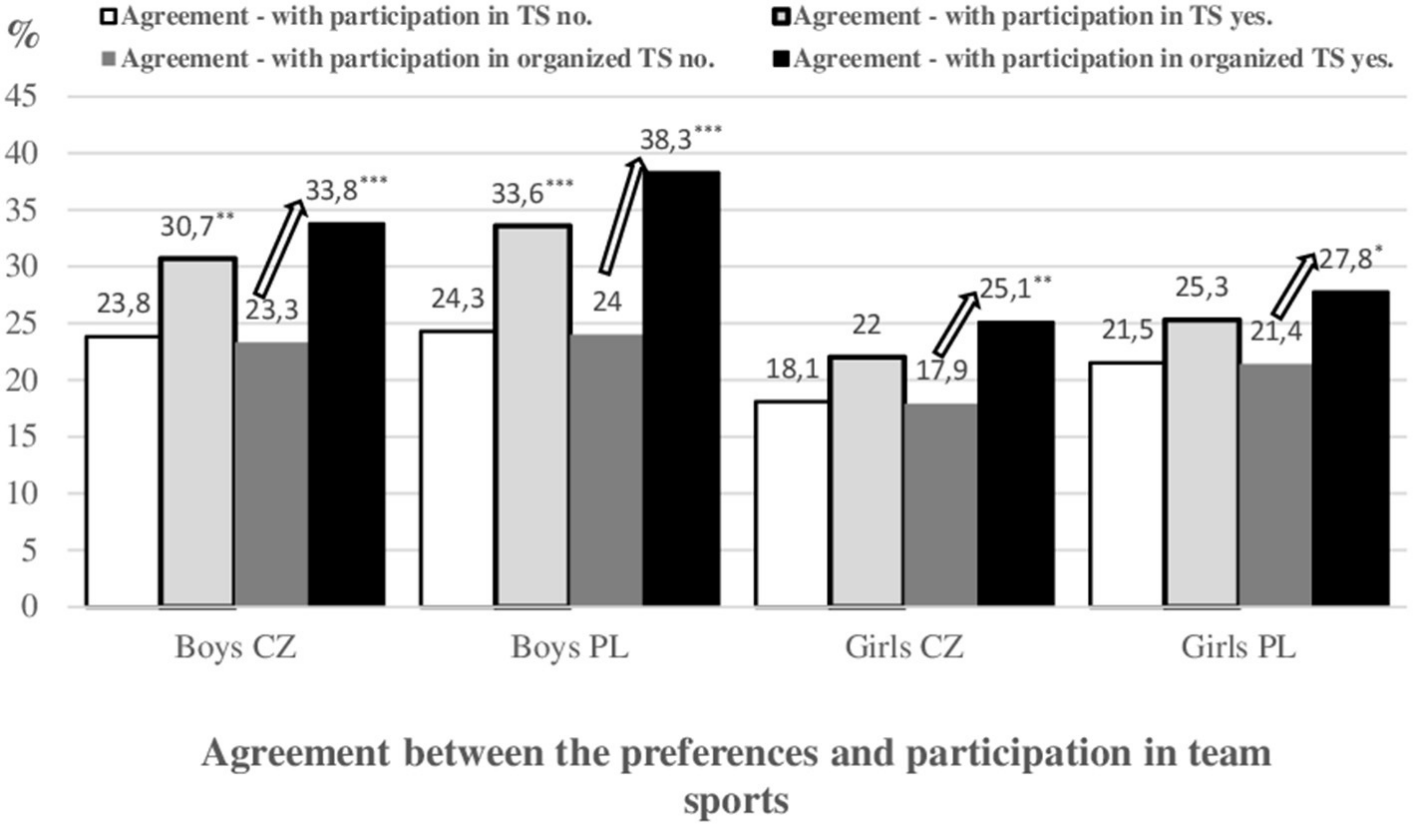
Meeting the weekly PA recommendations (at least 5 days and minimum of 60 min of MVPA/day and at least 3 days with at least 20 min of vigorous PA/day at week) by agreement between the preferences of team sports (TS) and participation in TS (yes–no) and by agreement between the preferences of TS and participation in organized TS (yes–no) (CZ—Czech Republic, PL—Poland).

Czech (χ^2^ = 9.51, *p* = 0.002, *r* = 0.136) and Polish boys (χ^2^ = 12.13, *p* < 0.001, *r* = 0.098) with an agreement between the preferences and participation in TS were significantly more likely to meet the weekly PA recommendations (at least 5 × 60 min of MVPA and at the same time, at least 3 × 20 min of VPA) than boys without an agreement (Figure 2). In girls, these differences were not statistically significant.

Czech boys (χ^2^ = 20.09, *p* < 0.001, *r* = 0.111), Polish boys (χ^2^ = 22.84, *p* < 0.001, *r* = 0.135), Czech girls (χ^2^ = 9.02, *p* = 0.003, *r* = 0.057), and Polish girls (χ^2^ = 4.48, *p* = 0.034, *r* = 0.053), with an agreement between the preferences and participation in organized TS, were significantly more likely to meet weekly PA recommendations than participants without an agreement.

### Meeting PA recommendations

Agreement between preferences and participation in TS increases the likelihood of achieving weekly PA recommendations in Czech and Polish boys and girls (Table 2). The agreement between the preferences and participation in organized TS increases the likelihood of achieving weekly PA recommendations in Czech and Polish boys and girls. The significant influence of the possibility of using cottages on the achievement of PA recommendations in Czech girls did not change the significance of the achievement of PA recommendations in either type of agreement. Participation in organized TS is the most important predictor of PA recommendations.

**Table 2:** Chances of achieving the 3 × 20 min of vigorous PA and 5 × 60 min of moderate to vigorous PA in boys and girls by agreement between the preferences and participation in team sports.

**Variables**	**Boys–CZ**		**Boys–PL**		**Girls–CZ**		**Girls–PL**	
	***OR* (95% *CI*)**	***p***	***OR* (95% *CI*)**	***p***	***OR* (95% *CI*)**	***p***	***OR* (95% *CI*)**	***p***
**Agreement between the preferences and participation in team sports**								
No ref. Yes	1.415 (1.134–1.765)	0.001	1.578 (1.220–2.042)	0.001	1.284 (1.001–1.646)	0.049		
**Cottage**								
No ref. Yes					1.284 (1.001–1.646)	0.001		
**Agreement between the preferences and participation in organized team sports**								
No ref. Yes	1.685 (1.340–2.120)	<0.001	1.958 (1.483–2.583)	<0.001	1.524 (1.150–2.019)	0.003	1.420 (1.025–1.968)	0.035
**Cottage**								
No ref. Yes					1.374 (1.132–1.667)	0.001		

OR, odds ratio; CI, confidence interval; CZ, Czech Republic; PL, Poland; p, significance level; Categorial covariates, Agreement between the preferences and participation in team sports (No–Yes), Agreement between the preferences and participation in organized team sports (No–Yes), Age (< 17 years or ≥17 years), Cottage use (No–Yes), Car use (No–Yes), Home (House or Flat), Dog ownership (No–Yes), Bicycle ownership (No–Yes).

It should also be considered that Czech (χ^2^ = 5.49, *p* = 0.019, *r* = 0.057) and Polish boys who preferred soccer (χ^2^ = 9.65, *p* = 0.002, *r* = 0.088) were significantly more likely to achieve PA recommendations (5 × 60 min of PA and 3 × 20 min of VPA) compared with those who did not. In contrast, no significant differences in the achievement of PA recommendations were observed between Czech (χ^2^ = 0.33, *p* = 0.567, *r* = 0.011) and Polish girls (χ^2^ = 3.41, *p* = 0.065, *r* = 0.046) who preferred volleyball and those who did not.

## Discussion

The results of the 14-year monitoring of PA preferences confirm the priority preference for TS among adolescents in the Czech Republic and Poland. The preferences for TS among Czech and Polish boys and girls have been stable over the long term and are consistent with the results of previous studies (31, 32). Higher TS preferences (62%) were observed by Zeng, Hipscher, and Leung (43) in high schools in the USA, but among younger 9–12-year-old adolescents. The most preferred TS among Czech and Polish adolescents is clearly soccer among boys and volleyball among girls, which corresponds with the popularity of soccer among boys (10, 44) and volleyball among girls (45, 46). The popularity of soccer is also reflected in boys' participation in soccer in high schools in the USA (47). From a global perspective, soccer is popular in all age groups and regions (48). Similar findings have been reported by numerous other studies; for example, younger adolescent boys in Turkey prefer soccer and basketball, whereas girls prefer basketball and volleyball (49). Basketball (57.9%) and soccer (21.9%) for boys, and basketball (31.0%) and volleyball (21.8%) for girls were among the top 10 reported activities among U.S. high school students (50). Globally, there has been an increase in the popularity of soccer among girls (51).

The results show that the preferences are significantly affected by gender in different types of sports (52, 53). Allison et al. (4) emphasized the need to focus on the participation of high school girls in TS in the context of health benefits. Kuśnierz et al. (54) called for respecting gender preferences in physical education lessons. Girls prefer “fun–pleasure–entertainment” forms of PA, while boys prefer “exercise–sweat–fitness” forms of PA in physical education lessons. There is a lack of knowledge concerning the extent to which social aspects prevail over fitness or health aspects in girls preferring TS. It is likely that many girls prefer volleyball for psychosocial reasons and for lower physical demands. Therefore, the focus should be on ways to decrease the differences between boys and girls in relation to fitness-oriented PA (32). Promoting new entertaining and more fitness-intensive simplified ways of playing volleyball is one option for supporting fitness. An extracurricular volleyball program has been shown to improve body composition in overweight adolescent girls (55).

Knowledge of adolescents' PA preferences is a key motivator for participation in PA in school and out-of-school environments. Providing opportunities for PA among adolescents should be based on their PA preferences (56). Therefore, the effort to reach an agreement between preferences and participation in TS is crucial, both for the development of TS and promotion of adolescents' PA. The indicator of agreement between preferences and participation in TS is important but insufficient because there are other motivational, environmental, health, performance, or psychosocial determinants that influence successful active participation in sports (57). For example, it turns out that building playgrounds for soccer and basketball is the best strategy for increasing the number of visitors and PA in parks (58). Johnson (59) emphasized the need to defend TS with respect to arguments that TS are not lifelong sports. However, concerning aspects of family life, simplified forms of TS may have significant intergenerational effects on sports education, as well as on a physically active lifestyle. For the effective use of TS, it is therefore important to connect school, club and leisure education of adolescents, and the symbiosis of institutional and family support (60). Monitoring the trends in preferred and pursued types of PA in adolescents and efforts to achieve agreement between them is essential for the effective promotion of PA in less active adolescents (46).

The agreement in preferring TS and participation in organized TS is significantly associated with a higher weekly PA in both Czech and Polish boys and girls. These results are especially relevant at a time of overcoming the negative effects of pandemic restrictions. It is precisely the decline in participation in organized PA associated with the decline in adolescent PA that is alarming in terms of health impacts (61).

A serious finding is that the agreement between the preferred and pursued TS significantly supports the achievement of the PA recommendations. The achievement of PA recommendations is consistent with other findings concerning the association between PA motives and PA types (62). In adolescent boys with different motivations, the degree of PA achievement ranged from 19 to 41%, whereas in girls, it ranged from 10 to 33%. It was also confirmed that an important moderator variable in the achievement of PA recommendations was active participation in organized PA. The results of this study correspond with the finding that boys and girls who prefer soccer are more likely to achieve PA recommendations than non-preferring individuals (44). Unfortunately, the IPAQ-long form questionnaire has limitations in assessing the level of recommended PA. Therefore, we believe that a more credible approach is to analyze intergroup comparisons in the achievement of PA recommendations.

In summary, the study verified a positive effect of consensus between preference and participation in TS on total weekly PA, on weekly vigorous PA, and on meeting PA recommendations. Therefore, we consider the results of the study to be valuable for the Central European region, but also for countries where TS is an important part of adolescent PA. The priority is to establish optimal settings for the realization of preferred TS and to ensure “equal” access to TS for as wide a range of adolescents as possible. It is important to create optimal settings for the implementation of preferred TS, particularly in organized forms of PA, where restrictions had the most negative impact during the pandemic (63).

Regarding other types of PA/sports, comparing the status and trends in TS in future research will require methodological unification of preferences, as well as detailed specification of preference indicators. Research on PA preferences should be performed using a holistic/comprehensive approach (64), taking into account numerous demographics, national-traditional, social, economic, health, educational, media, sports, and other aspects. Long-term monitoring of the status and trends in preference and participation in TS will also allow a detailed analysis of the negative effects of the pandemic on the types of PA and on the achievement of PA recommendations by adolescents.

### Strengths and limitations

The strength of this study is the 14-year monitoring of the status and trends in the preferences and participation in TS in different educational and sports environments in the Czech Republic and Poland. To our knowledge, the impacts/effects of the agreement between preferences and participation in TS on the achievement of weekly PA recommendations have not yet been subject to research. Similarly, continuous monitoring of the weekly PA structure before and during the pandemic is rare. The results of this study may have an international reach, especially in the context of the Central European region.

The greatest limitation of this study is the impossibility of ensuring a representative sample of participants in each year of the study. The yearly exchange of schools, regions, types of schools, and classes only decreases the sample's lack of representativeness. However, the almost identical results at each stage of the research convincingly characterize the status and trends in the preference for and participation in TS by adolescents in the Czech Republic and Poland. Another problem is the overall unclarity and entropy concerning the understanding, definition, and methods of examining preferences, popularity, wishes, inclination, and psychologically clearer attitudes toward PA. There are also different approaches for defining and understanding team sports and team PA.

Future research should focus on analyzing associations between PA preferences, adolescent age, gender, and participation in different types of PA.

## Conclusion

Based on 14-year monitoring of the status and trends in sport preferences and participation in the Czech Republic and Poland, TS is the most preferred type of PA in these countries. In TS, soccer is the most preferred PA for boys and volleyball for girls. The most important finding of the study is that consistency in preference and participation in TS is positively related to higher VPA and total weekly PA of adolescents. It was confirmed that following the status and trends in TS preferences when creating an environment for PA participation promotes increasing PA and achieving PA recommendations. Creating optimal settings for “equal” access for adolescent boys and girls to realize their preferred TS is also essential for school, municipal, and state sports policies. These policies should aspire as much as possible to increase active participation of adolescents in organized TS, as this is difficult to replace in acquiring and reinforcing the habit of regular PA for precisely those boys and girls who prefer TS.

## Data availability statement

The raw data supporting the conclusions of this article will be made available by the authors, without undue reservation.

## Ethics statement

Studies involving human participants were reviewed and approved by the Ethics Committee of Human Research of the Faculty of Physical Culture at Palacký University, Olomouc (no. 24/2012), and the Jerzy Kukuczka Academy of Physical Education in Katowice (no. 2/2008). Written informed consent to participate in this study was provided by the participants' legal guardian or next of kin. Written informed consent to participate in this study was provided by the participants' legal guardian/next of kin.

## Author contributions

KF, DG, and JM collected the data, secured funding, and conceptualized the manuscript. MK and PH have reviewed and edited the manuscript. All authors contributed to the article and approved the submitted version.

## Funding

This research was supported by the Czech Science Foundation—research projects: Objectification of comprehensive monitoring of school mental and physical strain in adolescents in the context of physical and mental condition (Grant No. GA 13-32935S), Multifactorial research of built environment, active lifestyle, and physical fitness of Czech youth (Grant No. GA 14-26896S), and Social Norms Intervention in the prevention of excessive sitting and physical activity promotion among Czech adolescents (Grant No. GA 17-24378S).

## Conflict of interest

The authors declare that the research was conducted in the absence of any commercial or financial relationships that could be construed as a potential conflict of interest.

## Publisher's note

All claims expressed in this article are solely those of the authors and do not necessarily represent those of their affiliated organizations, or those of the publisher, the editors and the reviewers. Any product that may be evaluated in this article, or claim that may be made by its manufacturer, is not guaranteed or endorsed by the publisher.
